# Use of Mustard Seed Footbaths for Respiratory Tract Infections: A Pilot Study

**DOI:** 10.1155/2020/5648560

**Published:** 2020-01-23

**Authors:** Katja Goetz, Aune Hinz, Jost Steinhäuser, Ulrich von Rath

**Affiliations:** ^1^Institute of Family Medicine, University Hospital Schleswig-Holstein, Campus Luebeck, Ratzeburger Allee 160, 23538 Luebeck, Germany; ^2^Hausarztpraxis im Hafenhaus, Zum Hafenplatz 1 (Skandinavienkai), 23570 Travemuende, Germany

## Abstract

**Objective:**

Respiratory tract infections (RTIs) are the most commonly treated acute problems in general practice. Instead of treatment with antibiotics, therapies from the field of integrative medicine play an increasingly important role within the society. The aim of the study was to evaluate whether mustard footbaths improve the symptoms of patients with RTIs.

**Methods:**

The study was designed as a pilot study and was carried out as an interventional trial with two points of measurement. Between November and December 2017, six practices were invited to participate. Two of them participated in the study. Patients were included who presented with an RTI at one of the involved primary care practices during February and April 2018. Participants in the intervention group used self-administered mustard seed powder footbaths at home once a day, to be repeated for six consecutive days. The improvement of symptoms was measured using the “Herdecke Warmth Perception Questionnaire” (HeWEF). A variance analysis for repeated measurements was performed to analyse differences between the intervention and control groups.

**Results:**

In this pilot study, 103 patients were included in the intervention group and 36 patients were included in the control group. A comparison of the intervention and control group before the intervention started showed nearly no difference in their subjective perception of warmth measured by the HeWEF questionnaire. Participants of the intervention group who used mustard seed footbaths for six consecutive days showed an improvement in four of the five subscales of the HeWEF questionnaire.

**Conclusions:**

This study could provide a first insight into a possible strategy to improve symptoms regarding RTI by using mustard seed footbaths.

## 1. Introduction

Respiratory tract infections (RTIs) are the most commonly treated acute problems in general practice [1]. The treatment of RTI often involves the prescription of antibiotics. However, as RTIs are mostly due to viral infections, antibiotics are not an appropriate treatment [2, 3]. Such unnecessary use of antibiotics is considered as a major risk factor for developing resistances in particular [4].

Therefore, different national and international initiatives exist to reduce the use of antibiotics in patient treatment. Consequently, the “German Strategy against Antibiotics Resistance” (Deutsche Antibiotika-Resistenzstrategie, “DART”) of the German Federal Government recognised the urgent need for an effective infection treatment which simultaneously restricts the use of antibiotics [5]. Furthermore, the World Health Organisation (WHO) strategy on traditional medicine supports the use of integrative medicine and strengthening the self-care process of patients [6].

A systematic review supports the effective use of integrative medicine, especially Chinese herbal medicines, for the treatment of RTI [7]. It has been shown that different biochemical processes are responsible for a positive effect, such as antiviral action, antipyretic action, and antiinflammatory action [7]. Similar effects have been found in the mustard plant, which contains glucosinolates, especially sinigrin. Different studies have shown its beneficial pharmacological effects against cancer, antibacterial, antifungal, antioxidant, antiinflammatory and wound-healing properties, and biofumigation [8, 9]. Mazumder et al. discussed that sinigrin is one of the glucosinolates whose bioactivity should be further explored and its known activity enhanced through optimal delivery to the human body [9]. Moreover, the combination of thermogenic substances like mustard and warm footbaths could have a beneficial effect on the perception of illness. As a complement, warm footbaths could improve the immune status [10].

Therefore, it can be assumed that mustard seed footbaths could be one option for reducing the symptoms of RTI. The aim of the current pilot study was to evaluate whether mustard footbaths improve the symptoms of patients with RTI.

## 2. Materials and Methods

### 2.1. Study Design

**Figure 1:**
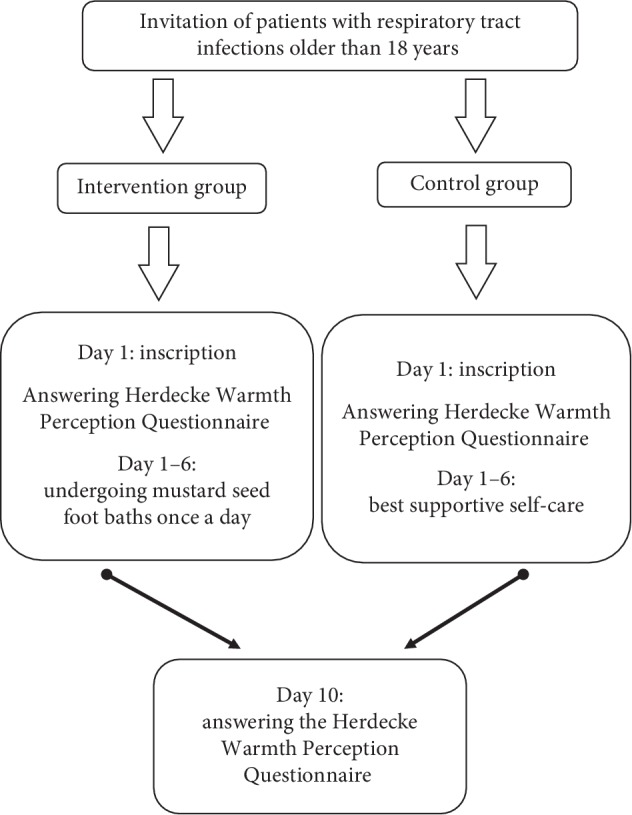
Procedure of the study.

The study was designed as a pilot study. Two points of measurements where assessed by the questionnaire, before (T0) and ten days later (T1) after intervention. Detailed information is shown in Figure 1.

### 2.2. Recruitment of Primary Care Practices and Patients

The recruitment of the primary care practices was based on personal contact. Between November and December 2017, six practices were invited to participate. The practices were located in the north of Germany, Lubeck. Two practices participated in the study. During February and April 2018, patients who presented in one of the involved primary care practices with an RTI where invited to participate in this study. Written informed consent was obtained. Adults (over 18 years) with an RTI who suffered from the symptom “cold feet” were included. Exclusion criteria were severe illness in immediate need of antibiotic treatment, ongoing immunosuppressive treatment or conditions, chronic obstructive pulmonary disease, serious renal failure (GFR < 45 ml/min), skin problems, and hypersensitivity to mustard seed.

### 2.3. Intervention

Participants in the intervention group used self-administered mustard seed powder footbaths with at home once a day, according to the given instructions, to be repeated for six consecutive days. A hot mustard seed footbath consists of a footbath at 40°C to which three table spoons of ground black mustard seeds have been added and stirred. The footbath must come above the ankle joints. It is applied for 7 minutes. Then, the feet are washed with warm water and calming/nurturing oil can be applied. At the start of the footbath, there is a quick sensation of warmth, a little burning sensation, and a slight tingly irritation of the skin. This feeling changes into a plateau of intense superficial heat. Moreover, the patients' feet grow warm, and they experience slight sleepiness and very often a feeling of well-being. The footbath is to be finished after seven minutes or just before the skin gets irritated and the strong smell and the burning sensation of the skin grows uncomfortable. After each footbath, participants are invited to lie down and take a 30 minute break without interruptions by television or mobile phones. Having applied a mustard seed footbath in the evening, the patient becomes sleepy and allows them to quickly and easily fall asleep with warm feet.

### 2.4. Measurements

The “Herdecke Warmth Perception Questionnaire” (HeWEF) was used for subjective ratings of warmth [11]. The questionnaire assessed sensations of the body warmth for up to 28 different items on a five-point rating scale, ranging from 0 “fully agree” to 4 “fully disagree.” These items were summarized to 5 scales by calculating the sum score. See Table 1 for a description of the 5 scales and the corresponding items. The questionnaire was validated and showed good psychometric properties [12]. Furthermore, the sociodemographic data of the participants were evaluated, such as age and gender. The reasons for recommendation for a mustard seed footbath were assessed by 7 possible reasons (e.g., common cold, cold feet, and chest cold).

### 2.5. Data Analysis

Data evaluation was carried out using the statistics programme SPSS 25.0 (Inc., IBM). Differences between the sociodemographic data of the control and the intervention group were analysed using Student's *t*-test for continuous variables as appropriate and the chi-square test for categorical variables. The HeWEF was analysed descriptively. Group differences for each item of the questionnaire were evaluated using the nonparametric Wilcoxon rank-sum test. Furthermore, a variance analysis for repeated measurements was performed. At first, the control and intervention groups were compared regarding the time and interaction (time × group) effect over two measurement points. In addition, the intervention and control groups were compared considering the time effect over two measurement points without consideration of the interaction effect. *P* ≤ 0.05 was determined as the significance level.

### 2.6. Ethical Approval

The study was approved by the Ethics Committee of the University of Luebeck, Germany (no. 17-286, 09th October 2017).

## 3. Results

The sociodemographic data of the intervention and the control group are listed in Table 2. Significantly, more female patients participated in the intervention group than in the control group (74.8% vs. 44.4%). Patients in the intervention group were significantly older than in the control group (49.9 years vs. 40.1 years). The main reason for participating in the intervention group was common cold (69.9%).

### 3.1. Herdecke Warmth Perception Questionnaire—Descriptive Analysis

Table 1 presents the descriptive data of the HeWEF questionnaire of the intervention and the control group before the intervention with mustard seed footbaths started. With the exception of 4 items such as “I freeze a lot,” “I tend to shiver a lot,” “I'm physically fine right now,” and “I'm psychologically fine right now,” no significant differences were found between the intervention and the control group regarding the HeWEF questionnaire.

### 3.2. Longitudinal Effects of Mustard Seed Footbaths in the Intervention and the Control Group

The longitudinal effects of mustard seed footbaths in the intervention and the control group are presented in Table 3. The variance analyses for repeated measurement showed a time effect in nearly all dimensions of the questionnaire with the exception of “need for warmth” (*F* = 0.07; *P*=0.79). No statistical significance was observed regarding the time × group effect. However, a nonsignificant tendency was found in the dimension “devotion” (*F* = 2.78; *P*=0.09) in favour of the intervention group.

### 3.3. Longitudinal Effects of Mustard Seed Footbaths

Analysing longitudinal effects, only subjects who had completed two measurements in the intervention group (*n* = 88) and in the control group (*n* = 30) were included in the variance analyses of repeated measurements and are shown in Tables 4 and 5. In these analyses, no interaction effects were considered. In the intervention group, significant improvements were observed for the dimensions “sensation of cold” (*F* = 20.15; *P* < 0.01), “devotion” (*F* = 15.4; *P* < 0.01), “exhilaration” (*F* = 5.89; *P*=0.02), and “unwellness” (*F* = 17.41; *P* < 0.01). With the exception of “unwellness” (*F* = 11.29; *P* < 0.01), no significant improvements were observed in the control group over two measurement points (Table 5).

## 4. Discussion

To our knowledge, there has been little research on the effects of mustard seed footbaths on the improvement of symptoms of RTI. Our results showed that more female patients were included in the intervention group. The patients of the intervention group were more than 9 years older than in the control group. Nearly, 15% of the participants in the intervention group stopped their participation in the intervention and did not respond to the questions after six days. It can be assumed that participation in such an intervention is acceptable and feasible. However, a further qualitative study will be helpful to evaluate the attitudes and experiences of participants who used mustard seed footbaths.

In this pilot study, the comparison of the intervention and the control group before the intervention showed nearly no difference in their subjective perception of warmth measured by the HeWEF questionnaire. For the participants of the intervention group who used mustard seed footbaths for six consecutive days, an improvement is observed in four of the five subscales of the HeWEF questionnaire. These are “sensation of cold,” “devotion,” “exhilaration,” and “unwellness.” The results of our study compare favourably with a study which showed that mustard footbaths increase the warmth perception of feet as measured using the HeWEF questionnaire [13]. Moreover, it can be assumed that mustard seed footbaths have a positive effect on the patients' well-being. Footbaths as a complementary treatment option have a positive impact on the immune function and on the patients' health due to its thermographic effect [10, 14]. It was also found that footbaths can lead to a reduction in stress [15]. Therefore, the relaxing effects of footbaths in combination with mustard plants could lead to a reduction in the perception of symptoms of RTI.

Different herbal preparations may be effective for the treatment of RTI or the common cold [16, 17]. Mustard, as a member of the Brassicaceae family, is amongst the oldest recorded spices. A review shows that mustard is used as a medicinal remedy for the treatment of different conditions such as bronchitis or diabetes [18]. Moreover, it was reported that it is used against colds and the flu [18]. Mustard plays an essential role in holistic herbal medicine, especially in Australia and New Zealand [18]. Nonspecific effects of treatment have to be considered in this context too. On average, up to 30% of an effect may be due to nonspecific aspects of care [19]. Future study designs should therefore control for this aspect.

### 4.1. Limitations

Despite the positive results reported here, our study has some limitations. The study was conceptualised as a pilot study with an explorative design. The presented results are therefore potentially strongly biased. A replication of this study with a larger sample size (intervention and control group) could help shed light on these matters and confirm the effects of footbaths with mustard seed in more detail. In view of the voluntary nature of participation in this study, which required a certain amount of motivational readiness on behalf of the subjects, we cannot generalise the results.

## 5. Conclusions

In spite of the mentioned limitations, this study could provide a first insight into a possible strategy to improve the symptoms of RTI by using mustard seed footbaths. The effect size of our pilot study was small. Therefore, further studies with slightly modified designs, especially randomised trials, are needed to establish the robustness of the possible effects of footbaths with mustard seeds.

## Figures and Tables

**Table 1:** Herdecke Warmth Perception Questionnaire—descriptive statistics.

Items of the Herdecke Warmth Perception Questionnaire^*∗*^	Scale^+^	Intervention group (*n* = 103) mean (SD)	Control group (*n* = 36) mean (SD)	*P* value
I freeze a lot	Sensation of cold	2.32 (1.21)	1.67 (1.31)	0.01
I tend to chill a lot	Sensation of cold	2.29 (1.26)	1.61 (1.23)	0.01
I always make sure I'm warm	Need for warmth	2.68 (1.01)	2.39 (1.15)	0.19
I often have cold hands	Sensation of cold	2.36 (1.26)	1.86 (1.44)	0.07
I often have cold feet	Sensation of cold	2.63 (1.29)	2.17 (1.50)	0.11
I tend to break out in sweats^#^	Need for warmth	1.50 (1.22)	1.25 (1.30)	0.20
I'm prone to hot flushes^#^	Need for warmth	1.40 (1.36)	1.14 (1.24)	0.36
I avoid cold rooms	Need for warmth	1.93 (1.24)	1.65 (1.18)	0.27
I prefer a well-warmed room	Need for warmth	2.55 (1.10)	2.25 (1.27)	0.31
Regardless of the outside temperature, I always dress warmly	Need for warmth	2.21 (1.26)	1.83 (1.16)	0.09
I can really get excited about certain ideas	Devotion	2.95 (0.97)	2.66 (1.06)	0.19
When I pursue a goal that is important to me, I can forget everything else around me	Devotion	2.68 (1.01)	2.53 (1.03)	0.38
I'd rather see myself as an active person	Exhilaration	3.04 (0.82)	2.83 (0.89)	0.23
I know the state of my bodily warmth	Devotion	2.43 (1.07)	2.67 (0.93)	0.29
I can devote myself completely to something that is close to my heart	Devotion	3.22 (0.73)	3.14 (0.85)	0.76
When I care about things, I always find ways to organize my time so that I can devote myself entirely to them	Devotion	2.88 (0.83)	2.97 (0.88)	0.45
I really enjoy trying out a lot of new things at the moment	Exhilaration	2.35 (1.11)	2.28 (0.97)	0.80
I have a lot of ideas that I still want to realize	Exhilaration	2.78 (1.04)	2.47 (1.06)	0.13
I'm physically fine right now^1^	Unwellness	2.95 (1.01)	2.50 (1.03)	0.02
I'm psychologically fine right now^1^	Unwellness	2.00 (1.23)	1.49 (1.15)	0.03
I'm relaxed right now^1^	Unwellness	2.35 (1.16)	2.09 (1.12)	0.25
I'm nervous right now	Unwellness	1.35 (1.21)	1.11 (1.04)	0.37
I'm in a sad or depressed mood right now	Unwellness	1.29 (1.20)	1.00 (0.96)	0.27
I'm tired and exhausted right now	Unwellness	2.55 (1.36)	2.53 (1.13)	0.69
I'm feeling rather discouraged right now	Unwellness	1.24 (1.32)	0.94 (1.07)	0.35

^*∗*^Range from 0 (fully agree) to 4 (fully disagree). ^+^For each scale a sum score was calculated. ^#^Marker items not considered for further analysis. ^1^Recoding of these items into the same direction comparable to the other items. SD, standard deviation.

**Table 2:** Characteristics of the study population (*n* = 139).

Variables	Intervention group (*n* = 103) *N* (%)	Control group (*n* = 36) *N* (%)	*P* value
Gender			
Male	26 (25.2)	20 (55.6)	<0.01
Female	77 (74.8)	16 (44.4)	
Age, mean (SD); range	49.9 (16.9); 18–85	40.1 (16.3); 18–78	<0.01
Reasons for recommendation^*∗*^			
Common cold	72 (69.9)	—	
Cold feet	28 (27.2)	—	
Chest cold/bronchitits	26 (25.2)	—	
Sinusitis	12 (11.7)	—	
Tension/headache	11 (10.7)	—	
Difficulties falling asleep	8 (7.8)	—	
Migraine	1 (1.0)	—	
Took antibiotics in the last 12 months to treat a respiratory infection	19 (18.4)	2 (5.6)	
Taking antibiotics after treatment with mustard seed footbaths	10 (9.7)	—	

^*∗*^Multiple responses possible.

**Table 3:** Longitudinal effects of mustard seed footbaths in the intervention and the control group.

Scale		Intervention group (*N* = 88) mean (SE)	Control group (*N* = 30) mean (SE)	Time effect *F* (*P* value)	Time × group effect *F* (*P* value)
Sensation of cold	T0	9.61 (0.46)	7.23 (0.79)	23.96 (<0.01)	0.69 (0.41)
	T1	8.07 (0.46)	6.23 (0.79)		

Need for warmth	T0	9.28 (0.37)	8.19 (0.63)	0.07 (0.79)	1.13 (0.29)
	T1	9.50 (0.41)	7.81 (0.69)		

Devotion	T0	13.93 (0.35)	13.87 (0.59)	10.60 (<0.01)	2.78 (0.09)
	T1	14.92 (0.37)	13.97 (0.63)		

Exhilaration	T0	8.23 (0.24)	7.81 (0.41)	4.08 (0.05)	1.41 (0.24)
	T1	8.64 (0.24)	7.81 (0.41)		

Unwellness	T0	13.21 (0.57)	11.33 (0.98)	61.41 (<0.05)	0.06 (0.81)
	T1	9.16 (0.61)	7.57 (1.04)		

SE, standard error. Statistical significance *P* < 0.05.

**Table 4:** Longitudinal effects of mustard seed footbaths in the intervention group (*n* = 88).

Scale	T0 mean (SE)	T1 mean (SE)	Time effect *F* (*P* value)
Sensation of cold	9.31 (0.44)	8.07 (0.45)	20.15 (<0.01)
Need for warmth	9.28 (0.37)	9.50 (0.41)	0.54 (0.46)
Devotion	13.93 (0.35)	14.92 (0.37)	15.40 (<0.01)
Exhilaration	8.23 (0.24)	8.64 (0.24)	5.89 (0.02)
Unwellness	13.21 (0.57)	9.16 (0.61)	17.41 (<0.01)

SE, standard error. Statistical significance *P* < 0.05.

**Table 5:** Longitudinal effects in the control group (*n* = 30).

Scale	T0 mean (SE)	T1 mean (SE)	Time effect *F* (*P* value)
Sensation of cold	7.23 (0.79)	6.23 (0.79)	3.91 (0.06)
Need for warmth	8.19 (0.63)	7.81 (0.69)	0.81 (0.37)
Devotion	13.87 (0.59)	13.97 (0.63)	0.03 (0.86)
Exhilaration	7.81 (0.39)	7.81 (0.41)	0 (1.0)
Unwellness	11.33 (0.91)	7.57 (1.07)	11.29 (<0.01)

SE, standard error. Statistical significance *P* < 0.05.

## Data Availability

The data used to support the findings of this study are available from the corresponding author upon request.
